# δEF1 upregulates CDK4 transcription via the E2-box element on the CDK4 promoter

**DOI:** 10.3892/etm.2013.1376

**Published:** 2013-10-31

**Authors:** FEN HU, KAI WANG, YUNFENG ZHANG, LIXIA GOU, MENGMENG LUO, XIUJUN ZHANG, SHUANG YANG

**Affiliations:** 1 College of Life Sciences, Hebei United University, Tangshan, Hebei 063000, P.R. China; 2 Department of Life Sciences, Tangshan Normal University, Tangshan, Hebei 063000, P.R. China; 3 Medical College of Nankai University, Tianjin 300071, P.R. China

**Keywords:** δ-crystallin enhancer factor 1, cyclin-dependent kinase 4, promoter, E2-box

## Abstract

The zinc finger-homeodomain transcription factor, δ-crystallin enhancer factor 1 (δEF1) has been identified as a regulatory factor involved in the promotion of breast cancer cell proliferation via the downregulation of p21 and the upregulation of cyclin-dependent kinase-2 (CDK2) and CDK4 expression. However, the molecular mechanisms underlying the regulation of CDK4 expression by δEF1 have not yet been elucidated. The present study demonstrated that the ectopic expression of δEF1 in MDA-MB-231 breast cancer cells significantly increased the activity of the CDK4 promoter. Deletion of the E2-box (CACGTG), which is located at position -197/-191 on the human CDK4 promoter, significantly attenuated the activation of CDK4 transcription by δEF1. In addition, a CDK4 promoter-M construct was generated via site-directed mutagenesis of the E2-box on the human CDK4 promoter. Luciferase assay showed that the activation of CDK4 promoter-M activity by δEF1 was markedly decreased compared with the CDK4-promoter-0.4k promoter. Knockdown of δEF1 using RNA interference resulted in the inhibition of CDK4 promoter activity. These observations suggest that δEF1 upregulates CDK4 transcription via the E2-box element on the CDK4 promoter.

## Introduction

Breast cancer is a leading cause of cancer-associated mortality in females worldwide and in excess of one million new cases of breast cancer are diagnosed annually (1). Breast cancer cell progression is a coordinated process that involves cell cycle dysregulation and a specific gene expression program to determine tissue identity. Cell proliferation, differentiation, senescence and apoptosis are cell cycle-dependent, and the basic regulatory mechanisms of cell cycle progression rely on a multicomponent system. At different phases, progression through the cell cycle is regulated by sequential activation and subsequent inactivation of a series of cyclin-dependent kinases (CDKs), whose activity depends on interactions with cyclins and cyclin-dependent kinase inhibitors (CDKIs) (2–4).

δ-crystallin enhancer factor 1 (δEF1), a member of the zinc finger-homeodomain transcription factor family (5), regulates gene expression to modulate cell differentiation and tissue-specific functions (6). Evidence has suggested that δEF1 is important in breast cancer tumor growth and metastasis (7). To control breast cancer cell proliferation, δEF1 downregulates p21 and concurrently upregulates the expression of CDK2 and CDK4 (8). However, the direct molecular mechanisms underlying the regulation of CDK4 expression by δEF1 have not yet been elucidated.

To address this issue, a series of different length and E2-box-mutated CDK4 promoter luciferase reporter genes were constructed in the present study. Luciferase assays were used to assess the effect of δEF1 overexpression and knockdown on the activity of the human CDK4 promoter. In addition, the effect of human CDK4 promoter E2-box (CACGTG) deletion on the activation of CDK4 transcription by δEF1 was investigated. The aim of the study was to evaluate the role of the E2-box on the CDK4 promoter in the promotion of CDK4 expression by δEF1.

## Materials and methods

### Cell culture

MDA-MB-231 cells (American Type Culture Collection, Manassas, VA, USA) were maintained in Dulbecco's modified Eagle medium (DMEM)-high glucose medium (Gibco-BRL, Grand Island, NY, USA) supplemented with 10% fetal bovine serum (FBS; HyClone, Thermo Fisher Scientific, Inc., Waltham, MA, USA), penicillin (50 units/ml) and streptomycin (50 mg/ml). The MDA-MB-231 cells were plated at a density of 5×10^4^ cells/well in 24-well plates for use in luciferase assays. This study was approved by The Ethics Committee of Hebei United University (Tangshan, China).

### Construction of plasmids

The generation of full-length δEF1 expression vectors (δEF1-pcDNA6B) was performed as described previously (6). The generation of δEF1-specific small interfering RNA (siRNA) expression plasmids (si-δEF1) was also performed as described previously (8).

The human CDK4 promoter sequence was obtained by polymerase chain reaction (PCR) from human blood genomic DNA and cloned into pGL3-basic vectors (Promega Corp., Madison, WI, USA) using the following primers: CDK4-promoter-1.2k (−1132), 5′-TTC**GAGCTC**GTGTTCTGG ACAGTGCTAAGTGC-3′ (forward); CDK4-promoter-0.7k (−710), 5′-TTG**GAGCTC**GTCACTGAGCCTGTTGGATT-3′ (forward); CDK4-promoter-0.4k (−378), 5′-TTG**GAGCTC**GCA GACAGGCTGAAAGAC-3′ (forward); CDK4-promoter-0.1k (−87), 5′-TTG**GAGCTC**TCCCAGTCGAAGCACCTCC-3′ (forward); and CDK4-promoter (+59), 5′-TGCAAGCTT TCACCCCCACCCTCACCAT-3′ (reverse; bold text indicates *Sac*I restriction enzyme sites). Mutagenesis of the E2-box in the human CDK4 promoter was performed using a QuikChange Site-Directed Mutagenesis kit (Stratagene Corp., La Jolla, CA, USA) with the following primers: 5′-GGG TTGTGGCAGCCAGTCA**AA**TGCCCGCGGC-3′ (forward) and 5′-GCCGCGGGCA**TT**TGACTGGCTGCCACAACCC-3′ (reverse).

### RNA extraction and semi-quantitative PCR

MDA-MB-231 cells were transiently transfected with δEF1-pcDNA6B or δEF1-specific siRNA expression plasmids in 24-well plates using Lipofectamine 2000 (Invitrogen Life Technologies, Carlsbad, CA, USA). At 24 h subsequent to transfection, the total RNA was extracted using TRIzol reagent (Invitrogen Life Technologies). A total of 0.5 μg total RNA from each sample was used for first-strand cDNA synthesis using Moloney murine leukemia virus (MMLV) reverse transcriptase (Promega Corp.). A specific transcript of δEF1 was amplified by semi-quantitative PCR using the following primers: 5′-GGCCCCAGGTGTAAGCGCAG-3′ (forward) and 5′-CGGGCAGGTGAGCAACTGGG-3′ (reverse). Verification of the expression level of δEF1 was performed using semi-quantitative PCR. GAPDH was used as an internal control.

### Luciferase assay

MDA-MB-231 cells were cotransfected with CDK4-promoter-1.2k, CDK4-promoter-0.7k, CDK4-promoter-0.4k, CDK4-promoter-0.1k or the mutant human CDK4 promoter construct CDK4-promoter-M in 24-well plates using Lipofectamine 2000 (Invitrogen Life Technologies). At 24 h subsequent to transfection, lysates were prepared and the luciferase activity was measured using a Dual-Luciferase Reporter Assay System (Promega Corp.) according to the manufacturer's instructions. Luciferase activity was normalized using the Renilla luciferase activity. The luciferase activity of the extracts was assessed 24 h subsequent to transfection using a Betascope analyzer (Betagen, Waltham, MA, USA).

## Results

### Overexpression of δEF1 increases human CDK4 promoter activity

**Figure 1:**
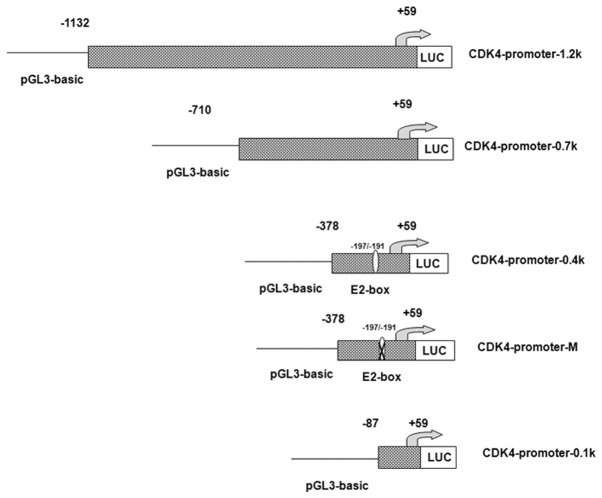
A series of different length and E2-box-mutated CDK4 promoters were cloned into the dual luciferase expression vector, pGL3-basic. These plasmids were known as CDK4-promoter-1.2k, CDK4-promoter-0.7k, CDK4-promoter-0.4k, CDK4-promoter-0.1k and CDK4-promoter-M, as shown in the figure. CDK, cyclin-dependent kinase; LUC, luciferase.

**Figure 2:**
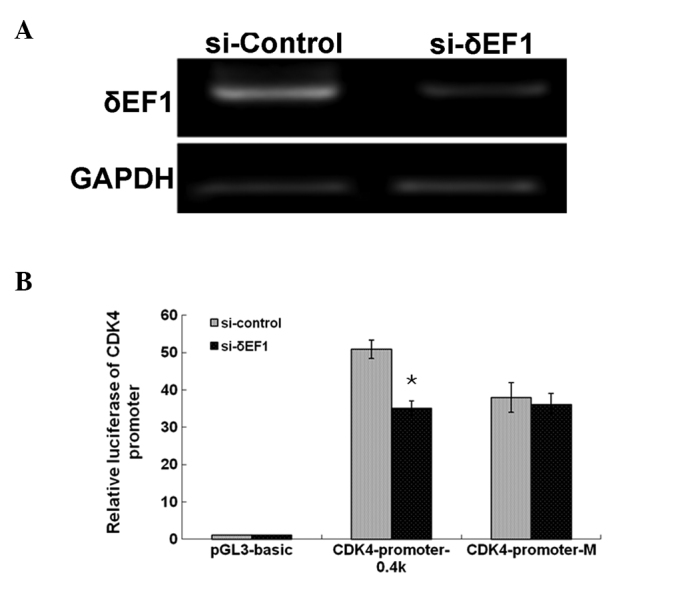
Overexpression of δEF1 enhances the activity of the CDK4 promoter. (A) Semi-quantitative polymerase chain reaction was performed to show δEF1 expression in the control and δEF1-overexpressing MDA-MB-231 cells. GAPDH was used as an internal control. (B and C) MDA-MB-231 cells in 24-well plates were cotransfected with CDK4-promoter-1.2k, CDK4-promoter-0.7k, CDK4-promoter-0.4k, CDK4-promoter-0.1k, CDK4-promoter-M promoter luciferase reporter (1 μg/well) and the δEF1 expression plasmid (1 μg/well), respectively. The luciferase activity of the extracts was assessed 24 h subsequent to transfection using a Betascope analyzer. Luciferase values are normalized with Renilla activities. ^*^P<0.05 in an unpaired Student's t-test when compared with the vector alone. Data represent three independent experiments. CDK4, cyclin-dependent kinase-4; δEF1, δ-crystallin enhancer factor 1; δEF1-pcDNA6B, full-length δEF1 expression vector.

In the present study, the CDK4 promoter region was amplified from human blood genomic DNA. Four different regions of the regulatory sequences in the CDK4 promoter, including ~1.2 kb of the upstream region, were cloned into the dual luciferase expression vector, pGL3-basic (Fig. 1). The overexpression of δEF1 mRNA was confirmed using semi-quantitative PCR (Fig. 2A). A comparison of the activity of these fragments in the dual luciferase reporter assays revealed that δEF1 overexpression significantly increased human CDK4 promoter activity of the CDK4-promoter-1.2k, CDK4-promoter-0.7k and CDK4-promoter-0.4k reporter genes. The increase was ~50% relative to the control (no δEF1 transfection) (Fig. 2B). This indicated that δEF1 was involved in the positive regulation of CDK4 transcription.

### δEF1 promotes the transcription of CDK4 through the E2-box on the CDK4 promoter

δEF1 has been reported to function as a transcriptional repressor by directly binding to the E2-box [CA(C/G)(C/G)TG] in the promoter region of target genes. δEF1 binds using its zinc finger clusters, which are located close to the N and C termini of the molecule (9,10). In the present study, a search using the transcription factor databases Transcription Element Search System (TESS; http://www.cbil.upenn.edu/cgi-bin/tess/tess. Accessed February 25, 2013) and TRANScription FACtor database (TRANSFAC; http://www.cbrc.jp/research/db/TFSEARCH.html. Accessed February 25, 2013) identified an E2-box (CACGTG) that is located at position -197/-191 of the human CDK4 promoter. In order to investigate whether δEF1 regulates the transcriptional activity of the human CDK4 promoter through this putative response element, a truncated CDK4 promoter reporter (CDK4-promoter-0.1k) was constructed (Fig. 1). The results showed that, by E2-box depletion, CDK4-promoter-0.1k exhibited no increased luciferase activity relative to the CDK4-promoter-0.4k (Fig. 2B). Furthermore, δEF1-induced transactivation of CDK4-promoter-0.1k was almost non-existent (Fig. 2B), indicating that deletion of the E2-box on the human CDK4 promoter significantly attenuated the activation of CDK4 transcription by δEF1.

The E2-box on the human CDK4 promoter was mutated to generate the CDK4-promoter-M construct as shown in Fig. 1. A luciferase assay demonstrated that the activation of CDK4-promoter-M promoter activity by δEF1 was markedly decreased compared with the CDK4-promoter-0.4k promoter (Fig. 2C). These data indicated that δEF1 promoted the transcription of CDK4 through the E2-box on the CDK4 promoter.

### Knockdown of δEF1 inhibits the promoter activity of CDK4

**Figure 3:**
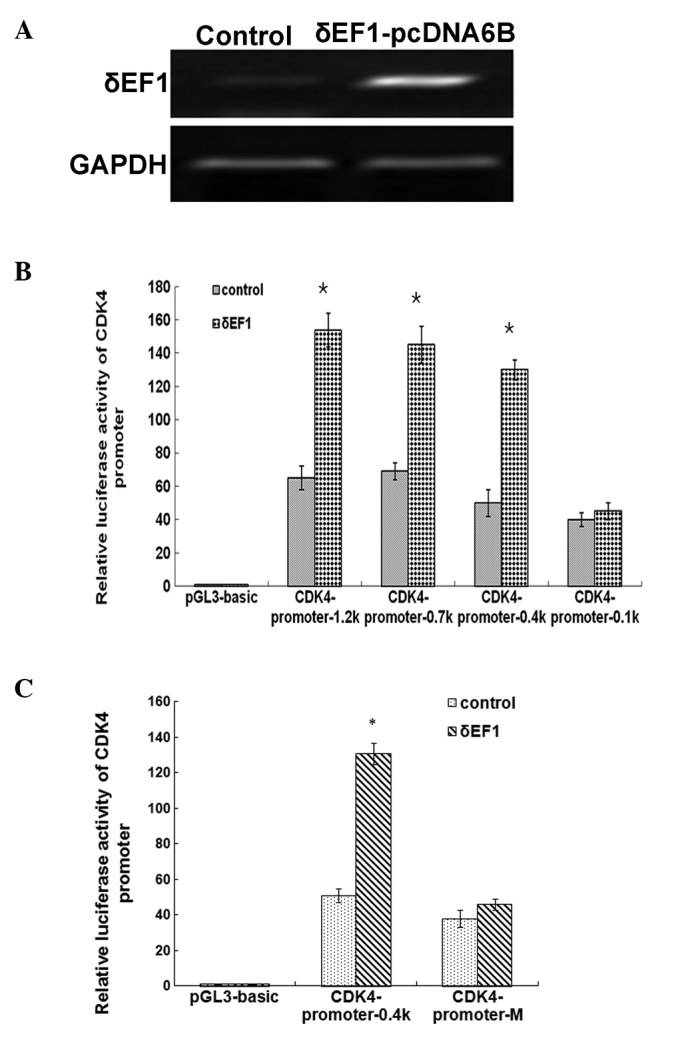
Knockdown of δEF1 inhibits the activity of the CDK4 promoter. (A) Semi-quantitative polymerase chain reaction was performed to show the efficiency of δEF1 knockdown in MDA-MB-231 cells. GAPDH was used as an internal control. (B) MDA-MB-231 cells in a 24-well plate were cotransfected with CDK4-promoter-0.4k, CDK4-promoter-M promoter luciferase reporter (1 μg/well) and δEF1-specific siRNA plasmids (si-δEF; 1 μg/well), respectively. The luciferase activity of the extracts was assessed 24 h subsequent to transfection using a Betascope analyzer. Luciferase values are normalized with Renilla activities. ^*^P<0.05 in an unpaired Student's t-test when compared with the vector alone. Data represent three independent experiments. CDK4, cyclin-dependent kinase-4; δEF1, δ-crystallin enhancer factor 1; siRNA, small interfering RNA; si-δEF1, δEF1-specific siRNA expression plasmid.

It was hypothesized that the knockdown of δEF1 using RNA interference was likely to result in inhibition of the CDK4 promoter. To test this, an siRNA expression plasmid targeting δEF1 or a scrambled control siRNA plasmid was cotransfected with the CDK4-promoter-0.4k reporter into the MDA-MB-231 cells. The knockdown of δEF1 mRNA expression was confirmed by semi-quantitative PCR (Fig. 3A). The results showed that δEF1 depletion resulted in significant inhibition of the promoter activity of CDK4 compared with the cells transfected with the control (Fig. 3B). Therefore, the downregulation of endogenous δEF1 in breast cancer cells is sufficient to allow inhibition of CDK4 expression.

## Discussion

A previous study focusing on the mechanism by which δEF1 promotes cell proliferation at the protein level have indicated a possible signal transduction pathway involved in this process (11). The aim of the present study was to further elucidate this mechanism at the transcriptional level. Investigations into the activity of the CDK4 promoter showed that δEF1 upregulated the activity of CDK4 promoter fragments (−1132 to +59 bp), (−710 to +59 bp) and (−378 to +59 bp), but not the fragment (−87 to +59 bp). These results indicated that the core promoter region of δEF1-enhanced CDK4 expression may be located within the (−378 to −87 bp) region of the CDK4 promoter. Knockdown of δEF1 using RNA interference resulted in inhibition of the CDK4 promoter. Furthermore, site-directed mutagenesis of the E2-box (CACGTG, −197 to −191 bp) on the human CDK4 promoter was performed and a luciferase assay demonstrated that δEF1 promoted the transcription of CDK4 by engaging the E2-box on the CDK4 promoter. This data indicate that the E2-box on the CDK4 promoter is important in the promotion of CDK4 expression by δEF1.

Traditionally, δEF1 has been identified as a widely expressed transcriptional repressor in a number of cellular processes, acting via interactions with corepressors or in competition with activators for DNA binding sites (6,12,13). However, a number of studies have indicated that δEF1 may also function as a transcriptional activator in the regulation of specific genes, including matrix metalloproteinase-1 (MMP-1) and ovalbumin (7,14). These findings were consistent with the results of our previous study, which showed that δEF1 activated MMP-1 transcription during breast cancer epithelial-mesenchymal transition (7) and induced micro RNA 21 (miR-21) promoter activity by binding to the E2-box on the miR-21 promoter (15). In addition, the results of the present study demonstrated that CDK4 expression was upregulated by the action of δEF1 on the E2-box of the CDK4 promoter.

In conclusion, the results of this study indicate that the E2-box on the CDK4 promoter is the core region in which δEF1 promotes CDK4 expression. These findings provide further insight into the mechanism of δEF1 gene-promoted breast cancer proliferation.
